# [Retracted] lncRNA ROR promotes the progression of renal cell carcinoma through the miR‑206/VEGF axis

**DOI:** 10.3892/mmr.2024.13229

**Published:** 2024-04-22

**Authors:** Jianguo Shi, Datian Zhang, Zhenhai Zhong, Wen Zhang

Mol Med Rep 20: 3782–3792, 2019; DOI: 10.3892/mmr.2019.10636

Following the publication of this paper, it was drawn to the Editor's attention by a concerned reader that certain of the Transwell cell invasion and migration assay data shown in Fig. 2D and F on p. 3786 were strikingly similar to data appearing in different form in other articles written by different authors at different research institutes, which had already been published. Owing to the fact that the contentious data in the above article had already been published prior to its submission to *Molecular Medicine Reports*, the Editor has decided that this paper should be retracted from the Journal. The authors were asked for an explanation to account for these concerns, but the Editorial Office did not receive a reply. The Editor apologizes to the readership for any inconvenience caused.

